# Prevalence, associated factors, and perceived causes of mental distress among visitors to holy water in Mekelle town, Tigray, North Ethiopia

**DOI:** 10.3389/fpsyt.2025.1563447

**Published:** 2025-10-22

**Authors:** Kenfe Tesfay Berhe, Tilahun Belete Mossie, Desalegn Massa Teklemichael

**Affiliations:** ^1^ Department of Psychiatry, College of Health Sciences, Mekelle University, Mekelle, Ethiopia; ^2^ Research Centre for Public Health, Equity and Human Flourishing, Torrens University Australia, Adelaide, SA, Australia; ^3^ Department of Psychiatry, College of Medicine and Health Science, Bahir Dar University, Bahirdar, Ethiopia; ^4^ Department of Epidemiology, College of Health Sciences, Mekelle University, Mekelle, Ethiopia

**Keywords:** mental distress, holy water, perceived causes, associated factors, Tigray

## Abstract

**Background:**

In low-income countries, mental disorders contribute to 12% of the global burden of disease compared to 8.1% in developed countries. Seeking help from religious institutions, which offer prayer and treatment with holy water, has been reported as a common strategy, particularly in developing settings. Despite the presence of many holy water sites in the study area, the problem has not been well studied. Therefore, this study aimed to assess the prevalence, associated factors, and perceived causes of mental distress among visitors to holy water sites in Mekelle town, North Ethiopia.

**Methods:**

A cross-sectional study design was employed, involving 380 participants in the quantitative study and 9 participants in the qualitative study, selected from three holy water sites in Mekelle town. Mental distress was measured using the Self-Reporting Questionnaire (SRQ-20). Data were analyzed with the Statistical Package for the Social Sciences (SPSS), version 20, and logistic regression analysis was used to determine associations at a p-value <0.05.

**Results:**

Among the 380 holy water visitors assessed, 147 (38.7%) had mental distress. Headache was the most commonly reported symptom, followed by feelings of fatigue and nervousness. The independent factors associated with mental distress were female sex (AOR = 1.68 (95% CI; 1.04, 2.72)), illiteracy (AOR = 3.2 (95% CI; 1.28, 6.91)), recent serious conflict with family members (AOR = 3.82 (95% CI; 1.89, 7.68)), and the belief that lack of faith causes mental illness (AOR = 2.33 (95% CI; 1.43, 3.79)).

**Conclusion:**

The prevalence of mental distress is high among holy water visitors compared to prior studies. This highlights the importance of engaging religious leaders and community members to integrate both religious and modern mental healthcare approaches.

## Introduction

Mental health is defined as “a state of well-being in which every individual realizes his or her own potential, can cope with the normal stresses of life, and is able to contribute to his or her community” (1). The World Health Organization (WHO) emphasizes that there is no health and development without mental well-being (2). Globally, approximately 25% of the population will develop a mental illness at some point in their lives. An estimated 450 million people suffer from mental or neurological disorders, and more than 150 million people suffer from depression. In low-income countries, mental disorders contribute to 12% of the global burden of disease, compared to 8.1% in developed countries (3).

Common mental disorders are major causes of morbidity and mortality worldwide, primarily affecting the working-age generation (4). Several studies have demonstrated that the incidence and prevalence of mental disorders in the working population have increased over the last few decades. These disorders affect 15%–25% of the global working population, impairing daily activities, social relationships, and financial income (4, 5).

A WHO study revealed that between 76% and 85% of individuals with severe mental disorders receive no treatment in low- and middle-income countries, compared to 35%–50% in developed nations (6). Almost half of the world’s population lives in countries where, on average, there is one psychiatrist to serve 200,000 people or more. Other mental healthcare providers trained in psychosocial interventions are even scarcer (6).

In developing countries, mental disorders are often not regarded as life-threatening problems and therefore lack adequate public health attention, as morbidity and mortality from malnutrition and infectious diseases remain very common (7, 8). A review of interventions for the treatment and prevention of selected mental disorders in low- and middle-income countries concluded that depression can be treated effectively in such settings with low-cost antidepressant medications or psychological interventions such as interpersonal therapy or cognitive behavioral therapy (9).

Mental distress is a mental disturbance that encompasses symptoms of psychiatric disorders that arise from stressful conditions. It is manifested by prominent symptoms such as sadness, frustration, and fearfulness, often accompanied by numerous somatic complaints (10). It is also characterized by unpleasant subjective states, such as feeling tense, worried, and worthless. These states can reduce emotional resilience and impair the ability to enjoy life or cope with pain, disappointment, and sadness (10, 11). Mental distress combines abnormal thoughts, emotions, and behaviors that significantly affect an individual’s lifestyle in areas such as self-efficacy, autonomy, competence, and the ability to realize one’s intellectual and emotional potential (1, 12). Individuals with severe symptoms often exhibit emotional instability, neglect of self-care, social isolation, poor communication, and frequent somatic pain (13).

Currently, mental distress and substance use are leading causes of disability and morbidity worldwide (10, 12). In member states of the European Union, the costs associated with mental distress and other neuropsychiatric disorders account for a 3%–4% loss of gross national product in each country (14). A community-based study conducted in Jimma town, southwest Ethiopia, reported a prevalence of 22.7% (15). Another study conducted in Addis Ababa, the capital city, and in the rural community of Butajira reported prevalence rates of 17.4% and 11.7%, respectively (16, 17).

A qualitative study in a southern Ethiopian community revealed that seeking help from religious institutions, which provide prayer and treatment with holy water, was a common strategy. Participants believed that through prayer and holy water, “the devil will scream and leave the patient.” Religious institutions, such as Christian and Islamic prayer sites, were frequently the first point where people sought help, followed by other options if no improvement occurred (18). Another community-based study in Ethiopia showed that rural residents were more likely to endorse traditional treatments, whereas urban residents tended to combine modern and traditional treatments (19).

There are positive findings regarding the benefits of religious affiliation among patients with mental illness. One study reported that religiously unaffiliated individuals had significantly higher rates of lifetime suicide attempts than those with a religious affiliation. It has also been recommended that psychotherapy is important when combined with religious support (20). Traditional treatment methods are more commonly preferred for addressing symptoms of mental disorders, whereas modern medicine is more often chosen for treating physical diseases or symptoms (21).

Studies suggest that Christianity perceives mental disorders as resulting from personal sin, lack of faith, or disobedience, and some churches advise against psychiatric medication. This highlights the need for continuous education to foster collaboration between Christian and mental health communities (22). The likelihood of experiencing mental distress is increasing as individuals are exposed to a variety of stressors in today’s life circumstances. Early alleviation of symptoms through psychotherapy is more cost-effective than the financial expenditure required once mental illness has advanced. However, in developing countries, many people spend considerable time with religious and traditional healers before seeking medical care, due to multiple challenges in the region.

Ethiopia’s current mental health strategy recommends collaborating with faith-based organizations to address these issues. This study aims to identify the magnitude of mental distress and its contributing factors. The findings will provide baseline information for all concerned stakeholders, especially mental health professionals, encouraging them to expand services in these areas, thereby improving the mental well-being of the community.

The causes of mental disturbance are heterogeneous, as reported in various studies. Religious and spiritual factors are increasingly being examined in psychiatric research to determine the extent to which individuals with mental health problems visit holy water sites and to identify mechanisms through which the mainstream health system can support them. The overall objective of this study was to assess the perceived causes, prevalence, and associated factors of mental distress among people visiting holy water sites in Mekelle.

## Methods and materials

### Study area

The study was conducted in Mekelle town, the capital of the Tigray region, located 783 km north of Addis Ababa, Ethiopia. The population was 323,700 in 2015. The town is divided into seven sub-cities. Approximately 91% of the population are Orthodox Christians, and 7.7% are Muslims (23, 24). There are more than 15 Orthodox churches in the town, and almost all of them provide holy water treatments. Among them, Saint Kidane-Mihret, Abune-Gebremenfes-Kidus, and Saint John churches are the three most well-known sites. The study was conducted at these three holy water sites.

Study design and period: A cross-sectional study design with both quantitative and qualitative components was conducted from December 2015 to June 2016. The source population included everyone who visited holy water sites in Mekelle, while the study population consisted of those who visited the selected sites during the data collection period. For the in-depth interviews, three priests who provided holy water treatment and nine participants (three men and three women) were purposely selected.

### Eligibility criteria

All individuals aged 18 years and older who provided written consent and could understand the interview process were included, as data collection was conducted by mental health professionals. Participants from Mekelle and its surrounding areas were eligible. However, individuals with serious medical illnesses or those who were acutely disturbed were excluded.

### Sample size and sampling technique

The sample size was calculated using a single population proportion formula with the following assumptions: a 95% confidence interval, a proportion of 22.7% (15), and a margin of error of 3%. The calculated sample size was 382, and after adding a 10% nonresponse rate, the final sample size was 420 subjects for the quantitative study. A total of 380 individuals ultimately participated, yielding a response rate of 90%. For the qualitative study, in-depth interviews were conducted with three priests who provided holy water treatment and six holy water followers (three men and three women) from the selected sites.

### Data collection procedures

A letter of invitation was sent to the responsible religious authorities and the regional health bureau. Recruitment flyers were posted in the compounds of the holy water sites. Data were collected by psychiatric nurses who were familiar with the local context and religious practices and experienced in psychiatric clinical work. Data collection was conducted in the morning, as the majority of clients receive holy water and attend religious prayer services at that time.

The Self-Reporting Questionnaire (SRQ-20) was used to detect mental distress. This instrument was developed by the WHO to screen for mental distress in primary healthcare and low-income community settings. The SRQ-20 is a first-stage screening tool and does not diagnose mental illness. It includes yes/no responses to items assessing mental distress over the past month. A cutoff score of six or more was used to screen for mental distress. This tool is widely developed and validated by the WHO and has been previously translated into Amharic and used in epidemiological studies in Ethiopia (25, 26).

Other structured questions were used to assess sociodemographic characteristics and related factors. Data collection and supervision were carried out by four psychiatric nurses. The data collectors and supervisor received one day of training from the principal investigator on the study instrument, consent procedures, confidentiality measures, and data collection techniques. An interviewer-administered method was used. The principal investigator and the supervisor checked the completed questionnaires for consistency and completeness each day.

The questionnaire was first forward-translated from Amharic to Tigrigna by native speakers proficient in both languages. This version was reviewed and compared with the original Amharic version and then back-translated into Tigrigna by other translators. Finally, discussions between the translators and the principal investigator resolved any inconsistencies, and the final version was produced.

For the qualitative study, a guide was prepared to conduct in-depth interviews that focused on perceived causes of mental illness. The findings from the qualitative study were triangulated with the results of the quantitative study. Mental distress was the outcome variable, while socioeconomic, demographic, psychosocial, substance use, and medical illness factors served as the independent variables.

Mental distress, defined as psychological and emotional disturbance, was identified as a score of six or more on the SRQ-20 tool.

### Data quality assurance

Data were collected by trained professionals. A pretest was conducted on a sample representing 5% of the total study population at a holy water site outside the study area. Based on the pretest results, some questions were modified for clarity. Data collection was carried out by psychiatric nurses who were familiar with the local context and religious practices and who had experience working as psychiatric clinicians. Regular supervision was provided by the supervisor and the principal investigator, both senior mental health experts. Each day, completed questionnaires were checked for completeness and consistency.

### Data analysis procedure

Data were coded, entered, edited, cleaned, and analyzed using the Statistical Package for the Social Sciences (SPSS), version 20. Dependent and independent variables were first entered into bivariate logistic regression one at a time to detect associations with the outcome variable. All variables associated with mental distress, and those with a p-value ≤ 0.2, were entered into a multivariate logistic regression model using the enter method (default) to control for potential confounders. Variables with a p-value < 0.05 in the multivariate model were declared significant predictors of mental distress. The findings were presented in tables and graphs. Model fit was checked using the Hosmer–Lemeshow and the likelihood ratio tests.

For the qualitative data, after transcription, similar themes were organized using thematic analysis and triangulated with the quantitative findings.

### Ethical considerations

Ethical clearance was obtained from Mekelle University, College of Health Sciences. Permission letters were secured from the clergy of the Orthodox Tewahdo Church, the Mekelle zone office, and the selected holy water church administrators. Detailed information about the study was provided to all participants before data collection, and written consent was obtained. Participants had the right to decline or withdraw from the study at any time. Confidentiality was maintained at all stages of data processing. Those who reported suicidal ideation or scored ≥6 on the SRQ-20 were referred to the nearest psychiatric clinic. Discussions were held with holy water providers regarding severely ill patients, with an emphasis on linking them to psychiatric clinics.

### Strengths and limitations of the study

A strength of the study was its use of the SRQ-20, which is globally accepted, standardized, and validated in Ethiopian settings. Both qualitative and quantitative aspects were assessed. One limitation was that seriously ill patients or those unable to communicate were excluded, which may have contributed to an underestimation of prevalence.

This study has several limitations that should be considered when interpreting the findings. First, the results cannot be generalized to the broader population experiencing mental distress, as prevalence was estimated only among individuals who visited holy water sites. Second, the data did not capture sufficient details about participants’ income, although employment status was included. This may have led to missing the potential confounding effect of income on mental distress. Finally, the cross-sectional design limits the ability to draw causal inferences between the examined factors and mental distress.

## Results

A total of 380 subjects attending holy water sites participated in the quantitative study, yielding a response rate of 90%. In addition, nine participants (three religious leaders and six holy water visitors) were involved in the qualitative study through in-depth interviews.

### Socio-demographic characteristics

The mean age of the participants was 30 years (± 12.8), with an age range of 18–90 years. The largest proportion (28.7%) was young adults aged 20–24. The majority of the participants were women (63.9%) and urban dwellers (81.6%). The majority (98.4%) identified as Tigrian by ethnicity, and 93.1% as Orthodox Christians.

With respect to education and occupation, 32.1% had attended a tertiary education, and 25.3% were students. More than half of the participants (55%) were single (**Table 1**).

**Table 1:** Sociodemographic characteristics of subjects who attended traditional healing with holy water in the city of Mekelle, North Ethiopia, June 2016 (n = 380).

Variable	Categories	Count	%	Mental distress		P-value
				Yes	No	
Age (in years)	<= 19	40	10.5	14	26	.271
	20-24	109	28.7	32	77	
	25-29	73	19.2	26	47	
	30-34	47	12.4	19	28	
	35-39	36	9.5	15	21	
	40-44	17	4.5	7	10	
	45-49	14	3.7	5	9	
	>= 50	44	11.6	29	15	
Residence	Urban	310	81.6	103	207	.061
	Rural	70	18.4	44	26	
Sex	Male participants	137	36.1	43	94	.026
	Female participants	243	63.9	104	139	
Religion	Orthodox	374	98.4	144	230	.49
	Muslim	6	1.6	3	3	
Ethnicity	Tigray	347	91.3	136	211	.533
	Amhara	33	8.7	11	22	
Educational status	No formal education	57	15.0	35	22	.247
	Can read and write	22	5.8	13	9	
	Primary	65	17.1	22	43	
	Secondary	114	30.0	36	78	
	Tertiary	122	32.1	41	81	
Occupation	Unemployed	63	16.6	39	24	.008
	Housewife	37	9.7	11	26	
	Daily Laborer	34	8.9	12	22	
	Government employee	64	16.8	13	51	
	Farmer	27	7.1	20	7	
	Merchant	32	8.4	13	19	
	Student	96	25.3	32	64	
	Pensioner	5	1.3	1	4	
	Others	22	5.8	6	16	
Marital status	Single	209	55.0	72	137	.05
	Married	143	37.6	58	85	
	Divorced	16	4.2	13	3	
	Widowed	12	3.2	4	8	

### Proportion of mental distress across different psychosocial factors

A total of 147 participants scored ≥6 on the SRQ-20, indicating a prevalence of mental distress of 38.7% (95% CI: 33.8%–43.2%). By related factors, the prevalence was highest among rural residents (62.8%), women (42.8%), and individuals with no formal education (61.4%). Among those living outside of their families, 32.2% experienced mental distress. Four of the five divorced participants (81%), half of those with a physical illness (50%), and one-third of participants with poor social support (33.3%) also had mental distress.

Overall, 23.7% of participants lived outside of their family (either alone or with relatives), 38.7% reported a lack of social support, and 11.6% had a chronic physical illness. Current substance use was reported by 2.4% (cigarettes), 2.5% (khat), and 5.5% (alcohol) of respondents, respectively. In addition, 2.1% of participants reported the death of a spouse, 14.5% reported the death of a parent or other close family member, and 1.8% reported experiencing sexual or physical violence. Recent residential change within the past year, serious family conflict, work-related stress, and academic stress were reported by 12.1%, 11.8%, 20.3%, and 8.4% of participants, respectively. All participants agreed that “mental illness can be caused by stressful life events (**Table 2**).

**Table 2:** Proportion of mental distress across psychosocial factors among holy water visitors in the city of Mekelle, North Ethiopia, June 2016 (n=380).

Factors		Mental distress				P-value
		NO		Yes		
		Number	Percent	Number	Percent	
Living arrangement	Alone	47	72.3%	18	27.7%	.692
	Family	172	59.3%	118	40.7%	
	Relative/Friend	14	56.0%	11	44.0%	
Social or Family Support	Yes	135	57.9%	98	42.1%	.018
	No	98	66.7%	49	33.3%	
Physical Illness	Yes	22	50.0%	22	50.0%	.677
	No	211	62.8%	125	37.2%	
Mental Illness in the family	Yes	24	49.0%	25	51.0%	.221
	No	209	63.1%	122	36.9%	
Cigarette smoking	Yes	1	11.1%	8	88.9%	.104
	No	232	62.5%	139	37.5%	
Khat chewing	Yes	2	20.0%	8	80.0%	.512
	No	231	62.4%	139	37.6%	
Alcohol drinking	Yes	11	52.4%	10	47.6%	.183
	No	222	61.8%	137	38.2%	
Family or Parental death	Yes	26	47.3%	29	52.7%	.104
	No	207	63.7%	118	36.3%	
Sexual/Physical Violence	Yes	2	28.6%	5	71.4%	.239
	No	231	61.9%	142	38.1%	
Death of a spouse in the previous year	Yes	3	37.5%	5	62.5%	.358
	No	216	61.0%	138	39.0%	
Change of residence in the previous year	Yes	25	55.6%	20	44.4%	.803
	No	207	62.0%	127	38.0%	
Serious Conflict in the previous year	Yes	16	35.6%	29	64.4%	.014
	No	217	64.8%	118	35.2%	
Work-related stress in the previous year (n=354)	Yes	39	50.6%	38	49.4%	.059
	No	174	62.8%	103	37.2%	
Academic stress in the previous year (n= 357)	Yes	17	53.1%	15	46.9%	.347
	No	197	60.6%	128	39.4%	

**Figure 1:**
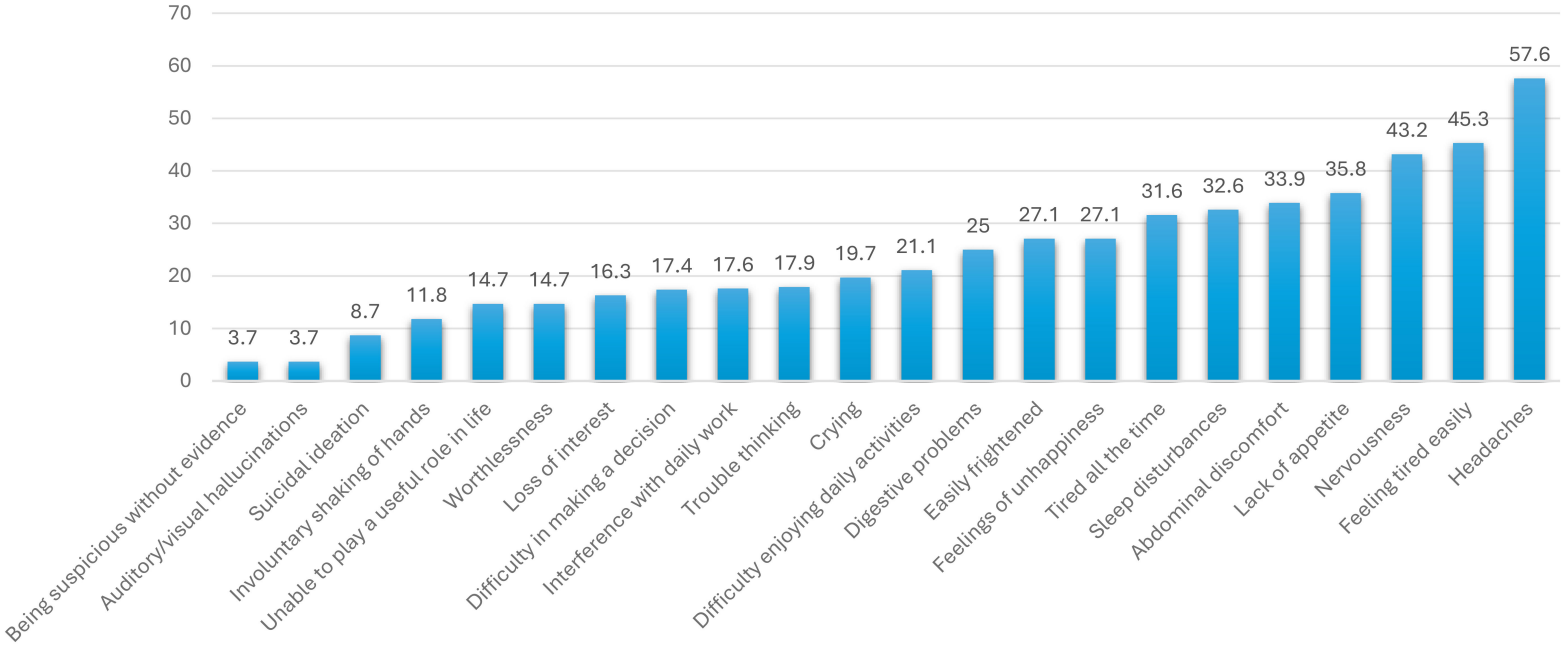
Distribution of people who attend traditional healing with holy water based on the reported symptoms of Mental Distress, city of Mekelle, North Ethiopia, June 2016 (n = 380).

Among the 20 symptoms screened, the most commonly reported were headache (57.6%), feeling easily tired (45.3%), and nervousness (43.2%). In addition to the SRQ-20 items, suspiciousness without known evidence and hallucinations (hearing voices or seeing images with no known stimuli) were each reported by 3.7% of participants (**Figure 1**).

#### Perceived causes of mental illness

Among the listed factors, traditional causes of mental illness were reported by more than half of the participants: lack of faith (63.7%), evil spirits (60.8%), and personal sin (55.3%). In contrast, scientifically supported causes were also frequently endorsed: substance use (83.2%), chronic illness (64.2%), and exposure to stressors (53.4%).

**Figure 2:**
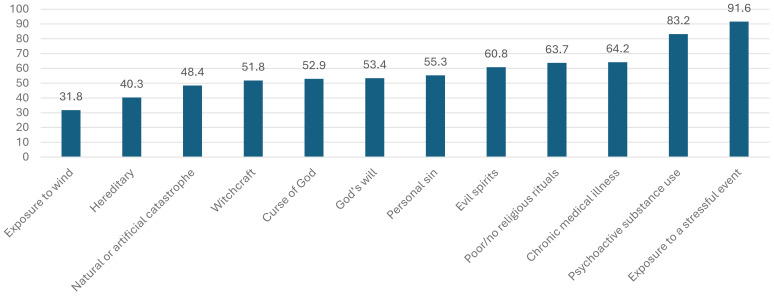
Percentage distribution of people who attended holy water treatments based on their perceived reasons for the causes of mental illness in Mekelle City, North Ethiopia, June 2016 (n = 380).

According to the qualitative data, nearly all participants reported believing in both modern (scientific) and traditional causes of mental illness. However, two participants distinguished between mental illness and an evil attack, stating: “*almost all participants believe in all types of the modern and the traditional perceived causes of mental illness. But, two participants said” mental illness and evil attack problem are different problems…. but, they are difficult to differentiate …. because the symptoms are almost similar…. but, shouting in the holy water is one symptom which help us that there is evil attack problem.*” (**Figure 2**). [SIC].

#### Other proximate issues related to mental distress

Approximately 86.3% of participants reported following holy water treatment at home, while 13.4% were admitted to a holy water site. In addition, 77% had sought other modes of treatment. Of these, 57.7% preferred modern treatment, while the rest used either traditional measures or a combination of traditional and modern approaches. The mean duration of follow-up was 61 days (± 345). From the qualitative findings, “*most participants believe only in the holy water treatment of mental distress, and even they don’t accept the combination of both holy water and medication…. why? They said, “Taking medication together with the holy water does show the individual’s poor belief of God’s power to treat from heart. One participant believed that “if the individual’s problem can’t be identified by Doctor holy water is good solution”* **[SIC].**

However, a 50-year-old priest disagreed with the above statement. He said, *“Combining both medication and religious help has no problem…. Mentally ill patient can be treated by holy water and medication…. Because God can use different mechanisms to help people…. that one mechanism could be using medication…. Medication is created with the willing and help of God. Example, Apostle Luke was a doctor and he treated many people at that time. So, we can’t say taking medicine is sin behavior.”* **[SIC]**.

Another priest emphasized that spiritual help is important alongside medical help. He said, “*A person who losses his money can be helped with advice from his friends or by professionals. But, for instance a person who involved in a sin activity and getting distressed because of it will be better helped by religious persons than professional counselors.”* **[SIC]**.

The reported reasons for visiting holy water were religious purposes (68.7%), physical illness (19.2%), and mental illness (12.1%). Among those who reported having a mental health problem, 19.6% of participants stated that they made no progress, while 34.8% and 45.6% reported good and very good progress, respectively. In the in-depth interview, all priests reported that mental distress had become the most common reason for using holy water.

#### Factors associated with mental distress

Variables with a p-value ≤ 0.2 in the binary logistic regression were selected for multivariate analysis. The final model revealed that a lower educational level was associated with mental distress: participants with no formal education and those who could read and write had approximately a threefold higher probability of developing mental distress compared to participants with a tertiary education or higher (no formal education, (AOR = 3.2, 95%CI; 1.28, 6.91)); (can read and write, AOR = 2.8(95%CI; 1.0, 7.07))).

Among psychosocial stressors, having serious family conflict in the previous year was strongly associated with mental distress. Participants who reported serious conflict had nearly a fourfold higher probability of developing mental distress (AOR = 3.82, 95% CI: 1.89–7.68). Among perceived causes, those who believed that a lack of faith could cause mental distress were more than twice as likely to develop mental distress compared to those who did not hold this belief (AOR = 2.33, 95% CI: 1.43–3.79).

Female participants were nearly twice as likely to develop mental distress compared to men (AOR = 1.68, 95% CI: 1.04–2.72) (**Table 3**).

**Table 3:** The degree of association between different factors and mental distress among individuals who sought traditional healing with holy water in the city of Mekelle, North Ethiopia, June 2016 (n= 380).

Variable		Mental distress		COR (95% CI)	AOR (95% CI)	P-value
		Yes	No			
Educational level	**No education**	**35**	**22**	**3.14(1.64, 6.03)**	3.24 (1.28, 6.91)**	**.003**
	**Can read and write**	**13**	**9**	**2.85(1.13, 7.23)**	2.78 (1.0, 7.07)**	**.004**
	Primary	22	43	1.01 (.54, 1.91)	.95 (.46, 1.98)	
	Secondary	36	78	.91 (.53, 1.57)	.83 (.44, 1.54)	
	Tertiary	41	81	1	1	
Sex	**Female participants**	**104**	**139**	**1.64 (1.01, 6.42)**	1.68 (1.04, 2.72)*	**.03**
	Male participants	43	94		1	
Living conditions	Alone	18	47	.49 (.19, 1.27)	.56 (.19, 1.68)	.176
	With family	118	172	.87 (.38, 1.99)	.91 (.34, 2.45)	
	With relatives/friends	11	14	1	1	
Marital status	Not married	89	148	0.88 (0.58, 1.35)	1.39 (.82, 2.35)	.158
	Married	58	85			
Physical illness	Yes	22	22	1.69 (.89, 3.17)	1.16 (.56, 2.39)	
	No	125	211			
Mental illness in the family	Yes	25	24	1.78 (.98, 3.26)	1.52 (.78, 2.98)	.263
	No	122	209			
Death of a parent or close family member in the previous year	Yes	29	26	1.96 (1.1, 3.48)	1.85 (.99, 3.47)	.052
	No	118	207	1	1	
Serious conflict in the previous year	**Yes**	**29**	**16**	**3.33 (1.74, 6.39)**	3.82 (1.89, 7.68)**	**.004**
	No	118	217	1	1	
Believe in witchcraft as a cause of mental illness	Yes	85	112	1.48 (.98, 2.25)	1.34 (.75, 2.40)	.178
	No	62	121	1		
Believe that mental illness can be caused by a curse	Yes	86	115	1.45 (.95, 2.19)	.78 (.43, 1.42)	.342
	No	61	118	1	1	
Believe that poor/no religious rituals cause mental illness	**Yes**	**107**	**135**	**1.94 (1.24, 3.04)**	2.33 (1.43, 3.79)*	**.006**
	No	40	98	1	1	
Believe that exposure to wind can cause mental illness	Yes	56	65	1.59 (1.02, 2.47)	1.6 (.87, 2.94)	.145
	No	91	168	1	1	
Believe that a catastrophe can cause mental illness	Yes	66	118	.79 (.52, 1.20)	.67 (.39, 1.17)	.193
	No	81	115	1	1	

*p-value<.05, **p-value<.005.

## Discussion

A total of 380 subjects participated in the quantitative study, and 9 subjects were included in the in-depth interviews. The largest proportion of participants were young adults aged 20–24 years, and 55% were single.

In this study, 147 of the 380 holy water visitors (38.7%) had mental distress. Headache was the most common symptom, followed by feeling easily tired and nervousness. Independent factors associated with mental distress included being a woman, having a low educational level, experiencing a recent serious conflict with family members, and believing that a lack of faith causes mental illness. These findings are consistent with those of other community-based studies conducted in Ethiopia. During the in-depth interviews, priests also reported that individuals experiencing either stressful conditions or behaviors they perceive as sinful tend to prefer religious help (15–17).

The proportion of holy water visitors with mental distress was higher than community-based prevalence rates. This may be because individuals with mental distress prefer religious help over modern treatment or use both religious and modern treatments (15–17). The prevalence of mental distress in this study was high (38.7%), similar to that reported in the Borana semi-nomadic population in southern Ethiopia four years ago, where participants commonly sought help at holy water sites first and then pursued other treatment options if no improvement occurred (18).

The prevalence of mental distress among holy water visitors in this study (38.7%) was lower than that reported in Saudi Arabia, where 95.6% of participants relied on reading the Holy Quran, 84.7% used blessed water, and 60.1% used blessed olive oil (22). However, it was higher than findings from South Africa, where 11% of participants consulted a religious or spiritual advisor (27). These differences could be attributed to variations in the perceived causes of mental distress across religions, cultures, and countries.

Studies in Nigeria and Tanzania reported comparable rates of mental distress, with 34.5% of participants seeking help in prayer houses and 48% at traditional centers, respectively (28, 29).

More than half of the participants in the current study attributed mental illness to traditional causes. Lack of faith, evil spirits, and personal sin were the three most commonly reported traditional perceived causes. Among scientifically supported factors, exposure to stressors was identified by nearly all participants and was also emphasized by qualitative respondents. Belief in the devil was particularly strong, with participants stating that *“*the devil will scream and leave the patient in holy water*.”* Many of the traditional causes of mental illness reported in previous studies from southern Ethiopia were also identified by over half of the participants in this study (19). Similarly, studies at faith healer sites in Nigeria and Saudi Arabia found that beliefs in magic, the evil eye, and stress were the primary perceived causes of mental illness (22, 28).

Among the holy water visitors in this study, only approximately half preferred modern treatment for their mental distress. Of the 38.7% identified as mentally distressed, only 12% reported visiting holy water specifically for mental distress-related help. This discrepancy may be due to participants not recognizing that their symptoms were related to mental distress, despite feeling unwell. Notably, 80% of participants who reported having mental health problems stated that they experienced good or very good progress in their mental distress after visiting holy water. This finding suggests that, because people commonly associate mental illness with causes such as the devil, personal sin, or lack of faith, working with priests to counsel patients with mild to moderate mental distress could provide meaningful benefits. Furthermore, previous studies have shown that religiously unaffiliated individuals had significantly higher rates of lifetime suicide attempts compared to those with a religious affiliation. Psychotherapy integrated with religious support has therefore been recommended to optimize outcomes for patients (20).

Approximately half of the participants in this study preferred traditional treatments over modern treatments in both the quantitative and qualitative findings. This preference may be influenced by personal and spiritual beliefs.

## Conclusion and recommendations

In conclusion, the present study provides insights into the link between holy water visitors and the level of mental distress. The study showed that the prevalence of mental distress is high among holy water visitors. Factors independently associated with mental distress were female sex, illiteracy, recent serious family conflict, and belief in a lack of faith as a cause of mental illness.

We recommend educating religious leaders and traditional healers to help them identify symptoms of mental distress and refer individuals who require psychiatric care. In addition, workshops involving religious leaders, traditional healers, and community leaders should be conducted to explore ways of integrating religious practices with modern mental healthcare. Active involvement from non-governmental organizations, universities, health bureaus, and the media is also essential for public education and to bridge the treatment gap between traditional and modern approaches. Finally, these findings highlight the importance of conducting further studies to clarify the impact of religious and traditional measures on the management of mental distress.

## Data Availability

The raw data supporting the conclusions of this article will be made available by the authors, without undue reservation.
